# Supplementary material for: Metabolic switching is impaired by aging and facilitated by ketosis independent of glycogen
Source: Aging (Albany NY). 2020 May 5;12(9):7963–84. doi: 10.18632/aging.103116 (PMC7244089; doi:10.18632/aging.103116)
Supplement: Supplementary Methods [file aging-12-103116-s001..pdf]

SUPPLEMENTARY METHODS

Additionally, a glucose tolerance test (GTT) was administered during the 7th week of dietary intervention. While the glucose injection did result in increased circulating glucose expression over time ($F_{[6,186]}$ = 34.09; $p < 0.001$; Supplementary Figure 1A), there were no differences across age ($F_{[1,31]}$ = 0.14; p = 0.71) or diet groups ($F_{[2,31]}$ = 1,15; p = 0.33), and none of these variables significantly interacted ($p \geq 0.10$ for all comparisons). Furthermore, there were no differences across age ($F_{[5,1]}$ = 0.28; p = 0.60) or diet ($F_{[5,2]}$ = 2.76; p = 0.08) in the AUC (Supplementary Figure 1B). However, injecting glucose did alter circulating levels of BHB differentially across diet groups ($F_{[2,31]}$ = 17.30; $p < 0.001$), and diet group significantly interacted with the amount of time post injection ($F_{[12,186]}$ = 4.55; $p < 0.001$; Supplementary Figure 1C). While there was a significant effect of time point for all groups ($F_{[6,186]}$ = 31.08; $p < 0.001$), there were no differences in BHB response across age groups ($F_{[1,31]}$ = 17.30; p = 0.06) nor did age interact with time point ($F_{[6,186]}$ = 1.54; p = 0.17). However, age did significantly interact with diet group ($F_{[1,31]}$ = 3.46; p = 0.04) such that there was a strong trend for aged free-fed rats to demonstrate lower levels than their diet-matched young counterparts ($F_{[1,8]}$ = 4.91; p = 0.06), whereas aged KD-fed ($F_{[1,11]}$ = 3.25; p = 0.10) and aged SD-fed rats ($F_{[1,12]}$ = 1.31; p = 0.28) did not differ from their diet-matched young counterparts. Finally, there were no differences across age ($F_{[5,1]}$ = 0.25; p = 0.62) or diet ($F_{[5,2]}$ = 0.63; p = 0.54) in the AUC (Supplementary Figure 1D).
