# Supplementary material for: Metabolic switching is impaired by aging and facilitated by ketosis independent of glycogen
Source: Aging (Albany NY). 2020 May 5;12(9):7963–84. doi: 10.18632/aging.103116 (PMC7244089; doi:10.18632/aging.103116)
Supplement: Supplementary Figure 1 [file aging-12-103116-s002..pdf]

SUPPLEMENTARY FIGURE

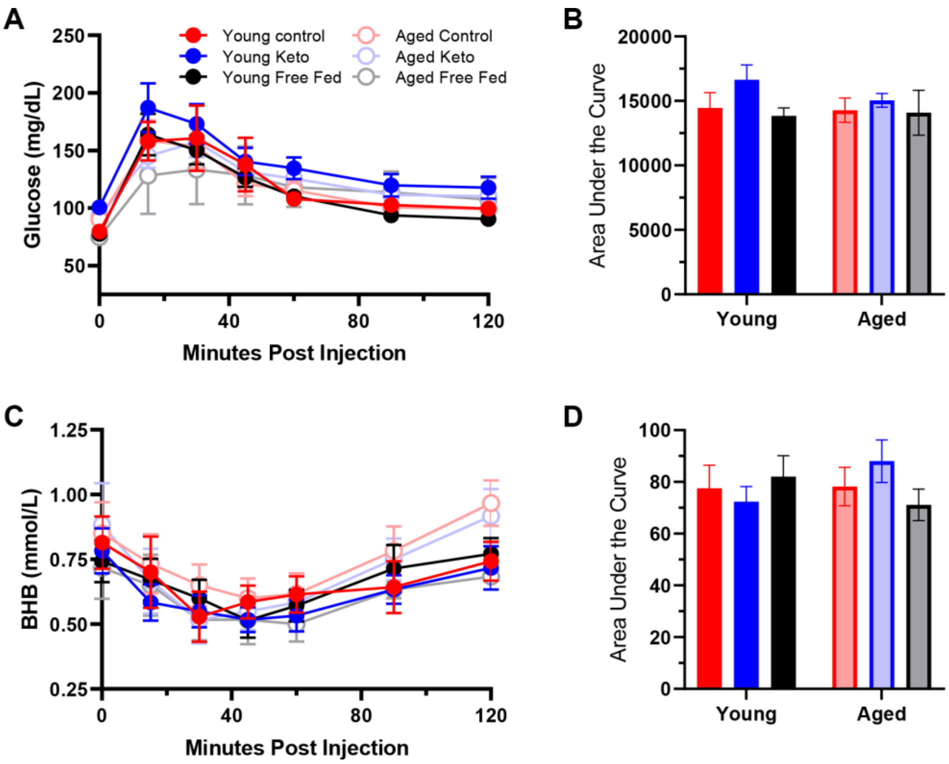

Supplementary Figure 1. Glucose tolerance testing (GTT) following 7 weeks dietary implementation. (**A**) There were no differences in (**A**) glucose values over time nor (**B**) area under the curve (AUC) across age or diet groups following an injection of a bolus of glucose intraperitoneally. However, diet did significantly affect (**C**) BHB values over time, (**D**) but no differences across age or diet groups were observed in the total AUC following glucose injection. Data are represented as group mean ±1 SEM.
